# Elderly self-management: a qualitative study

**Published:** 2010

**Authors:** Maryam Ravanipour, Shayesteh Salehi, Fariba Taleghani, Heidar Ali Abedi

**Affiliations:** * School of Nursing and Midwifery, Bushehr University of Medical Sciences and The Persian Gulf Tropical and Infectious Disease Research Center, Bushehr, Iran; ** Associate Professor, School of Nursing and Midwifery, Islamic Azad University, Khorasgan branch, Isfahan, Iran; *** School of Nursing and Midwifery, Isfahan University of Medical Sciences, Isfahan, Iran

**Keywords:** Self-management, elderly, grounded theory, qualitative research

## Abstract

**BACKGROUND:**

The population of elderly in Iran and in the world is increasing. It is predicted that the population of elderly reaches to 10 millions in Iran by the year 2019. Elders more than other age groups are at risk of chronic diseases and health problems; and elderly affects their self-management and makes them feel disabled. Since the knowledge of self-management for Iranian elderly is not well developed, this paper aimed to determine the concept of self-management for Iranian elders.

**METHODS:**

This was a qualitative study with grounded theory approach on Iranian elderly self-management. Data were collected through deep interviews with 26 participants in a period of one year and were analyzed using a Strauss Corbin analysis method.

**RESULTS:**

Self-management in the context of power means using different managing methods in dealing with daily life needs, especially in interactions with others in a way that accelerates affairs with efficiency and satisfaction. The main categories emerged from this qualitative study included: managing plans, managing life goals and policies, persuading the desired goals, managing self-care, directing others, coordinating and consulting with others.

**CONCLUSIONS:**

The findings of this study provided a deep understanding of elderly perceptions of self-management in their lives. These findings can be a baseline for future researches on developing effective health interventions such as developing a nursing model for increasing the elderly self-management abilities in Iran. Such a model can provide a strong basis for nursing care.

Having a long life has been a dream for human beings and in all ages and centuries, there has been constant efforts for survival and fight against death and disease in various ways. Getting old in a healthy condition is the right of every human being and this increases the significance of elderly and preventing its problems.^1^ The population of elderly is growing in the world. It is estimated that the population of people aged 60 years and higher is about 600 millions in the world and it will reach to a number in 2050 that for the first time in the human history, will be higher than children under 14 year old.^2^ According to the 1994 consensus Bureau in Iran,, about 6.6% of the total population of 65 million people and 7.2% of the total population of more than 70 million in 2004, are elderly aged 60 years and higher and it is predicted that by the year 2019 the number of elderly people in Iran reach ten million people.^3^,^4^

Aging is a gradual process which involves continuous evolution, maturation and adaptation at all levels and aspects of life. Above 60 years of age is usually known as elderly age.^5^ Unfortunately, quality of life does not increase with the quantity of life. Philosophy of health during life is one way of answering to the challenge of creating a world in which empowerment for all people regardless of their living conditions is considered normative and natural.^6^

One of the health challenges during elderly age is the concept of power and powerfulness and although there are many researches on the concept of power,^6^–^12^ few studies can be found on this concept in the elderly. Since people’s perception of power is based on their experiences from interaction with environment and others, use of qualitative methods (grounded theory) to proceed this topic sounds appropriate. Therefore, in a great project, the concept of power from the viewpoint of elderly was studied using grounded theory method. From among the basic structures forming this concept, self-management was resulted as the central concept that connected other concepts to each other.^13^

It seems that self-managing is a holistic idea. Wilson says that we need to study the fundamental incentive of self-management process in health care more closely. It is necessary to facilitate the empowering and contribution to the health care of customers to be able to strengthen self-management in individuals and relationships of institutions with power. On the other hand, this task requires customers’ incentive to self-management process.^14^ Based on this, the present research have sought elder’s perspective of the concept of self-management during this period of life. Therefore, this study aimed to achieve a deep understanding of the concept of self-management in elderly Iranian to be able to accordingly develop health care and strategies for empowerment that fit with the culture.

## Methods

Considering the objectives of the study that was to investigate the concept of self-management from the more general concept of power in individuals’ social interactions and processes, the grounded theory method of qualitative research was used. Grounded theory is a qualitative research method to explore social processes in human interactions and its roots can be found in the interpretive traditions of symbolic interaction.^15^ In this study, a total of 26 participants were interviewed with semi-structured deep interviews. The criteria to select elderly included aging over 60 years old and being able and willing to explain their experiences. The primary samples were selected from accessible elderly and continued based on the evolution of a theory that controls and guides the required features of next events and incidences for better development of theory. This sampling was stopped after saturation of all obtained categories and when no new meaningful property was produced. Each session of the interview lasted between 30 to 90 minutes (a mean of 45 minutes) based on the tolerance and interest of the participants. Most participants were interviewed two or more times and in total, 45 interviews were conducted. The interviews were conducted in the participants’ usual life environment and usually in their houses or various social environments. According to the results of the primarily obtained codes and constant analysis of the special comparison of this study, sampling was done in other environments such as elderly houses and also children and spouses of elderly. The effort was to choose people with maximum variety of economic, social and educational backgrounds as well as widows, married, with and without children. After obtaining their written consent and getting permission for recording interviews, a deep semi-structured interview was started with open questions such as “explain a usual day of your life”, “how do you do your personal tasks?” or “how do you face obstacles of doing your routines?” and continued based on the answers and in relation with self-management concept. At the end of each interview, it was transcribed and data were analyzed. All interviews, observations and hand writings were coded and analyzed based on Strauss and Corbin data analysis technique. In the first stage, transcribed interviews were read several times so that the researcher can come up with a kind of general understanding of the concepts in them. Then, the text were reviewed line by line and special parts of them that referred to a specific concept or directly what the interviewees had explained or the researcher implied the concept from it, were written as open code in the margin of the text. Out of more than 2400 primary open codes, those who were similar or had overlaps were revised to one refined specific code and finally 180 refined codes from the open primary codes were resulted which became the basis for final concepts.

In axial coding, similar coded data were put in one category, with the same title or a more abstract code that could contain them. Then, the differentiation of categories was checked for assurance. The codes that seemed having overlap were reviewed again and the codes were revised and were again categorized. In the third stage of coding, selective coding, the researcher sought for a category or central variable that could relate other variables and become the background to build the theory, while specified the process of investigating concept shaping. If there was ambiguity, complementary interviews were conducted. Moreover, the data collected by observations and notes by interviewees were used as sources. It must be noted that the process of assessing validity and reliability in qualitative studies is different from quantitative studies. In grounded theory method, the process of data analysis is constant comparison analysis, which increases the validity and reliability of data. Using this process, the researcher compares any new data with the previously analyzed data and unrelated data are filtered. Also, checking findings by returning them to informants to approve them helps increasing their validity. Audit is a better criterion for qualitative research compared to reliability.^16^ To determine the validity of data, people’s checking for the accuracy of implications, revision by colleagues and maximum variation sampling was used.^17^

### 

#### Ethical Considerations

Participants were completely aware of the study objectives and secrecy of their names. To record interviews, written consent was taken from participants and they were assured of being allowed to leave study at any time they wanted.

## Results

This study included a group of elderly who were relatively healthy and had no serious disability, living in the society. From their viewpoints, in spite of their awareness of various changes in their lives and their roles, they would not be able to achieve power and ability except that they use their self-management ability and methods to manage their goals, plans, coordination and guidance of resources and finally coping with problems. This elderly group was using self-management methods and strategies in various forms when talking or behaving and interacting with others. Some of these strategies were developed consciously and in some cases they were totally unconsciously chosen and based on the backgrounds and beliefs.

### 

#### Managing Goals

All participants in this study had one or more goals in their lives and they were trying seriously to achieve their goals and had even their philosophy of life. Most of them mentioned having a good life with a good end for both themselves and their children in a way that God and people would be happy with them. In other words, living healthy in all moral, social and physical dimensions was the headline of their lives. One of the elder participants wrote: “The correct determination of strategies and recognizing the correct way of passing through problems and removing obstacles to achieve goals while feeling power and control brings pride for the individual and the family and society.”

#### Program Managing

Most elderly mentioned routine and usual programs when describing their daily life. All elderly were participating in parts of house works and managing house works, and in cases were doing the financial management and expenses of life. One of them said: “Although our economic situation is not that well, I survive with the low income.” Most elderly had schedules for their daily life and even if they were not able to do them all, they were managing the schedule and consider it as one of the strong symbols of their power at home. Some had short term or long term plans for their lives and were active to achieve their plans by managing problems and dealing with obstacles.

#### Following Up Goals with Hope

In addition to having goals and planning for life, elderly who participated in this study considered trying to achieve goals as one of the most important tasks of life for a capable individual and considered this characteristic as a high value. One of the elders knew successes and experiences of life effective in creating hope for future and future successes. This elder said: “Hope is very effective in life and it is different from having expectations. I think having hope for future along with predicting problems and strategies to overcome problems is symbol of capability in elderly. If one uses his experiences in life, I don’t think he lost hope by aging. Because when he thinks he can see that he has been successful and can predict to be successful in future. If one knows his way well, it is impossible to lost hope and spirit. It happens a lot to me that I am physically unable to do something, but I have not lost the ability to think it over and finally I find the solution by thinking.”

#### Self Care Management

This category was obtained from joining two sub-category of health management and self care ability.

#### The Importance of Health Management

By aging and its effects on all body organs, gradually diseases and discomforts emerged in people and most of them had a chronic nature. Participants in this study by conscience referral to this fact and the importance of keeping healthy and preventing disease exacerbation, based on their language, beliefs and information, had specific method of life to keep healthy as much as possible. One of the participants said: “When your body gets weak, it affects your mind and you cannot think well and decide. For this reason, and because I have high blood pressure, I try to avoid salty food to keep as healthy as possible.” At the end of the interview, this participant asked the researcher to check her blood pressure, which proved her health.

#### Self Care Ability

Most elderly, in spite of their knowledge about emerging of various disabilities especially in their body, constantly try to have activities and take care of their personal tasks. An elder said: “Although I have pain in my legs and cannot walk for a long distance, I try to walk to the street and take a taxi to go shopping.” Another lady said: “I have osteoporosis and pain in my legs, but still do most of light works around house. I don’t like to impose my personal tasks on anybody.”

#### Guiding Resources

Elderly were giving guidance and advices to others based on their years of experiences. An elder said: “Even though all our children are living with their families, we are not neglecting them and frequently are in touch with them and if they have a problem, we two think together to find solutions for them. They usually accept our suggestions because they have seen good results.” Of course having scientific and professional skills for what they were doing and also experience and being skillful in that field and having control over situation were factors mentioned as prerequisites for being able to correctly guide others.

#### Coordination

Elderly who participated in this study consider themselves as being experienced and based on their experiences and the insight they had in managing life, they considered all dimensions of problems and situations to achieve their goals in the best and most proper way. A gentleman said: “Not only me, but everybody should study around what they are going to do and see if it is useful and profitable, what kind of budget is needed, have the exact accounting information for it, estimate everything and focus their control on the work to avoid wasting their money and follow up the work and control it until it is accomplished.”

It can be concluded that self-management in the background of feeling capable means that to use management methods in life tasks and especially in relation with others, in a way that accelerates works with appropriate outcome and satisfaction. Elderly mainly manage various resources they have access to, to evaluate the conditions, plan and coordinate resources and if they face obstacles in their way, they find coping methods to control the situation and reach internal satisfaction from their activities in daily life management.

## Discussion

As it was explained, having a goal and philosophy of life, managing aims and plans in life, knowing all dimensions of problems and trying to guide and coordinate life tasks are components of the self-management concept which were very important according to elderly point of view. From the participants’ viewpoints, capability and management of wishes and goals and finding best ways to access them considering situations and accessible resources is a kind of ability that every individual develops during life and various experiences and the more efficient they use this capability, the more power they have in life.

Process of self-management is an individual oriented process. Self-management is about customer oriented empowerment and people’s involvement in managing their life and the chronic conditions of their disease.^14^ If the person perceive a threatening situation manageable, it will have positive consequences for him.^18^ Successful management of life is certainly coordination of activities in work with family scopes. Of the indicators of successful management of life are life satisfaction, positive emotions, mental health and not being lonely.^19^ The elderly participants in this study directly or indirectly expressed satisfaction and capability in managing their life and achieving their goals, particularly managing their disease conditions and maintaining self-care; because they were fully aware of being at risk for diseases and more difficulties in elderly age.

Self-management is an ability that people need to manage their resources in a way that they can achieve their health goals. To have a successful elderly age, we need a self-management that is surpassed resources in an environment where lack of resources and reduction of them often occurs. In modern societies, the importance of self-management is very high. Learning self-management interventions to help compatibility with losses reduces the costs of the health system. With high levels of self-management ability, the elder is better able to maintain independence and authority in his life for a longer time. Most self-management interventions are focused on teaching adjustment with specific problems of the age such as depression, loneliness, increased risk of falling, insomnia or chronic diseases (in elderly age), while many elderly suffer from a combination of problems in various scopes of their lives, which causes creation of infirmity elderly.^20^

In today’s progressive modern world that is specified with fast changes, it is necessary to compliance policies and philosophy of life with role structures that are rapidly changing and environmental duties.^21^ Many factors influence self-management decisions. Economic status, cognitive status and capacity of sensitivity to competition demands such as receiving care and impaired recognition of self-management behaviors are issues related to elderly people that have an effect on their self-management.^22^ Having a positive mental framework, being self efficient, starting things by oneself, investing on resources for long term profitability, paying attentions to different sources, paying attention to multiplicity of resources^20^,^23^ are other effective factors on people’s self-management that were found more or less in the elderly talks. For example, they mentioned knowing different sources and investing on them and they supported the role of thinking in the efficient and effective management to a high extent.

## Conclusions

The results of this study reminded us that elders are aware of their internal environment (their conditions) and their external environment and health is particularly important for them. Therefore, considering the use of available actual and potential resources, they plan for their life to achieve the significant goals of their lives. Therefore, this can be a positive point in planning for health care of elderly and base various nursing interventions especially elderly health care models on this to improve the quality of life and health of elderly.

The authors declare no conflict of interest in this study. Ethical committee approved the study.
